# Complicated Acute Heart Failure Subsets

**DOI:** 10.1007/s11897-018-0402-z

**Published:** 2018-06-26

**Authors:** Prithwish Banerjee

**Affiliations:** 1 Department of Cardiology, University Hospitals Coventry & Warwickshire, Clifford Bridge Road, Coventry, CV2 2DX UK; 2 Faculty of Health & Life Sciences, Coventry University, Coventry, UK; 3 University of Warwick Medical School, Coventry, UK

**Keywords:** Acute heart failure, Renal failure, VT ablation, Ischaemic acute heart failure

Acute decompensated heart failure (ADHF) is a difficult clinical problem with poor outcomes. High in-hospital mortality and readmissions remain a concern despite advancement of treatment options for heart failure [1, 2]. The presence of complicating factors such as ventricular arrhythmias, renal dysfunction, or ischaemic heart disease (with or without hibernating myocardium) makes the management more complex and the outcomes bleaker [3–5]. However, newer treatment options continue to appear and further research continues. A focused look at these complicated ADHF scenarios is therefore helpful in assessing where we currently are in tackling these syndromes and what is just around the corner. Such focus is also likely to lead to new targeted thinking towards better avenues of management.

The previously dreaded ventricular arrhythmias in chronic heart failure have been managed much more effectively since the introduction of the implantable cardioverter–defibrillators (ICDs) [6]. However, recurrent VT despite optimal medical and cardiac resynchronisation therapy remains a problem with the affected patients occasionally experiencing recurrent shocks amounting to an electrical storm that can be very distressing to the patients [7]. Catheter ablation for ventricular tachycardia has come a long way but can it be used safely and effectively in such patients, especially those with refractory VT in ADHF? Dimitropoulos G and coauthors have, in their review, provided all the evidence available on VT ablation in both chronic and acute heart failure settings which clearly shows VT ablation to be an effective tool in ADHF but it needs to be tested further in larger trial settings.

Renal dysfunction in a heart failure patient is common and cardiorenal syndrome is now an established term. ADHF often brings with it worsening renal function and acute kidney injury that can raise concerns about the use of the much needed intravenous diuretic therapy as well as demand adjustment in ACE inhibitors, angiotensin receptor antagonists, or sacubutril valsartan [5]. Progressive renal dysfunction during a hospital admission with ADHF invariably prompts a referral to the renal physicians for renal support including dialysis. The review by Bielecka-Dabrowa A and coauthors highlights important new treatment options for future consideration using additional hypertonic saline with intravenous diuretics and tolvaptan which conserve renal function while still effecting adequate diuresis. Emerging markers for assessment of prognosis and the role of dialysis in this setting are discussed.

Although research involving hibernating myocardium is almost exclusively confined to the stable chronic heart failure population, the heart failure clinician frequently encounters it in ischaemic acute heart failures with a troponin rise. Ryan and Perera highlight the importance of considering coronary angiography if appropriate and where possible in ADHF for better management planning. We are also informed of the important REVIVED-BCIS2 trial, a UK multicentre study that randomises ischaemic heart failure into revascularisation by percutaneous intervention or optimal medical treatment and incorporates hibernating myocardium.

The reviews in this series on complicated acute heart failure syndromes bring up exciting future directions in managing these difficult subsets which often overlap. Tackling these subsets effectively is likely to go a long way in reducing mortality and hospitalisation rates.
